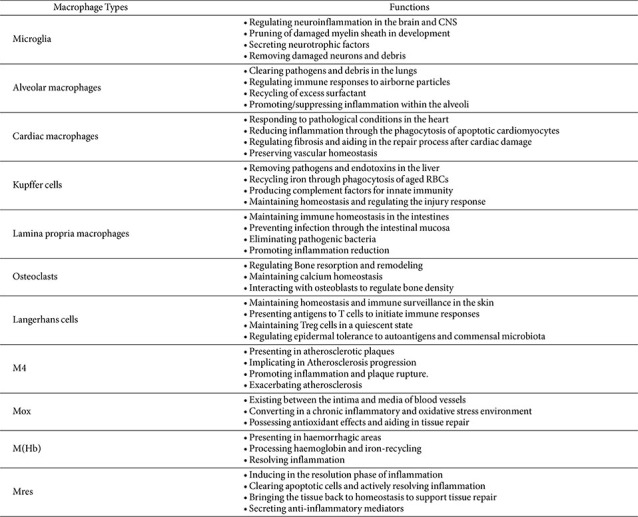# Macrophage Immune Responses in Viral Infections: Functional Plasticity and Therapeutic Targeting

**DOI:** 10.4014/jmb.2602.02035

**Published:** 2026-05-14

**Authors:** Ju Kim, Haneul Lee, Yong-Suk Jang

**Affiliations:** 1 Department of Molecular Biology and the Institute for Molecular Biology and Genetics, Jeonbuk National University, Jeonju 54896, Republic of Korea; 2 Department of Bioactive Material Sciences and Research Center of Bioactive Materials, Jeonbuk National University, Jeonju 54896, Republic of Korea

**Keywords:** Immune modulation, Macrophage, Pathogen, Therapy, Viral infection

## Abstract

Macrophages play dual roles, contributing to both protection and disease progression during viral infections. As crucial immune cells against pathogens, they activate innate immunity by releasing antiviral agents and inflammatory cytokines. At the same time, they serve as targets for infection and as carriers of viruses, thereby contributing to viral dissemination. This review emphasizes macrophage plasticity and the functional shifts it undergoes during M1/M2 polarization. It also explores mechanisms that maintain persistent viral reservoirs in chronic infections such as SARS-CoV-2 and discusses the impact of macrophage dysregulation on the immune microenvironment. Furthermore, it highlights recent advances in macrophage-targeted therapies, including reprogramming macrophages for homeostasis, eliminating pathogenic macrophages, and boosting antiviral responses.

## Introduction

Macrophages exhibit diversity among sub-lineages based on their origin and microenvironment. They are mainly classified into resident tissue macrophages (RTMs), which derive from embryonic progenitor cells, and monocyte-derived macrophages (Mo-Macs), which develop from circulating monocytes originating from myeloid hematopoietic stem cells (HSCs). RTMs differentiate into tissue-specific macrophages, such as microglia in the brain or Kupffer cells in the liver, and reside long-term within tissues (Table 1). Disruption of pathways involving the RUNX1 transcription factor, which is crucial for RTM differentiation, results in RTM deficiency and leads to the collapse of immune homeostasis within tissues [1, 2]. Monocytes circulate in the blood before migrating to sites of inflammation or tissue damage, where they differentiate into macrophages. This process helps replace or replenish the RTM pool. That is, Mo-Macs migrate to inflamed or injured tissues, exist temporarily, and require ongoing replenishment. Even within the same tissue, RTMs and Mo-Macs exhibit heterogeneity in their responses to microenvironmental stimuli. As tissue-specific macrophages, RTMs exhibit plasticity and are primarily involved in waste elimination, homeostasis maintenance, and tissue remodeling [3, 4]. Conversely, Mo-Macs mainly infiltrate during acute inflammatory states, performing roles such as clearing pathogens, responding to tissue damage, resolving inflammation, or inducing fibrosis. Unlike RTMs, they may also contribute to pathological processes in tumors or chronic infections [5].

Macrophages activated by various stimuli and signals are generally polarized into M1 (classical, pro-inflammatory) and M2 (alternative, anti-inflammatory) macrophages. M1 macrophages, primarily induced by IFN-γ and LPS stimulation, secrete pro-inflammatory cytokines, possess potent microbial/tumor cell killing capabilities, and the ability to promote Th1 responses [6, 7]. Conversely, M2 macrophages, induced by Th2 cytokines such as IL-4/IL-13, primarily produce anti-inflammatory cytokines and participate in tissue repair, fibrosis, the resolution of inflammation, and defense against parasites. They are further divided into specific subpopulations at the genetic and transcriptional levels, such as M2a, M2b, M2c, and M2d [8, 9]. In vivo, M1 and M2 macrophages coexist and can flexibly switch their polarization states over time in response to changing microenvironmental stimuli [10]. Furthermore, M1 macrophages are vital in anti-cancer and anti-microbial responses not only through the secretion of inflammatory cytokines such as IL-6, IL-12, and TNF-α, but also by producing high levels of nitric oxide (NO) and reactive oxygen species (ROS) and enhancing their ability to present antigens, thus performing defensive functions during acute infection and early tumor development [11]. Conversely, M2 macrophages suppress inflammation and support tissue regeneration by secreting growth factors, such as IL-10 and TGF-β, as well as various molecules that facilitate remodeling. However, in tumors, they show a dual role by promoting angiogenesis, contributing to immunosuppression, and helping form metastasis niches [12-14]. In chronic inflammatory and degenerative immune-mediated diseases such as rheumatoid arthritis (RA), atherosclerosis (AS), and Alzheimer's disease (AD), M1/M2 imbalance and alterations in RTM/Mo-Macs composition have also been reported to be associated with pathogenesis. Modulating specific polarization states is proposed as a potential therapeutic strategy [15, 16]. Particularly within the tumor microenvironment, macrophages tend to adopt an M2-like, immunosuppressive phenotype [17]. As a result, various immunotherapy strategies aimed at reprogramming or depleting them are currently in development.

Macrophages exhibit significant heterogeneity across tissue types and polarization states (M1/M2), leading to distinct outcomes in various diseases and viral infections. This review emphasizes the antiviral response of macrophages targeted early in viral infection, as well as the mechanisms viruses use to evade it. It also highlights recent research trends in macrophage-targeted therapies that exploit macrophages' dual functions.

### Macrophage Responses Within Virus-Driven Immune Microenvironments

In viral infections, macrophages play a key role in detecting pathogens, activating the innate immune response, and priming T cells [18]. When viruses or viral PAMPs come into contact with macrophages, macrophages rapidly produce type I interferons (IFN-β, IFN-α) through Toll-like receptor (TLR) signaling. This stimulates the activation of natural killer (NK) cells and T cells, as well as the expression of antiviral genes [19-21]. In this process, key signaling axes include the phosphorylation of interferon regulatory factor 7 (IRF7) and the activation of the ER-adaptor protein STING, which directly inhibit viral replication by inducing type I IFNs following cytoplasmic DNA recognition [22]. IFN-γ not only directly suppresses viral protein expression and replication, but also amplifies macrophage antiviral actions, such as NO production, through inducible nitric oxide synthase (iNOS) induction. Inflammatory cytokines such as IL-1β and IL-6 also contribute to antiviral responses by activating the MAPK/ERK pathway. However, viruses try to evade or block these pathways to keep infections going [23].

Various viruses have evolved strategies to evade macrophage antiviral functions by targeting the STING-IRF-IFN axis [24]. For instance, dengue virus (DENV) encodes the NS2B3 protease, which cleaves STING, thereby blocking the STING-dependent IFN response. In contrast, herpes simplex virus (HSV) inhibits Type I IFN production by preventing IRF activation mediated by the STING signaling complex [25, 26]. Furthermore, the TRIM family of proteins has been reported to play a crucial role in STING activation. TRIM29, expressed in alveolar macrophages (AMs), acts as a negative regulator of the antiviral immune response by modulating STING activation [27]. TRIM29 expression is upregulated by viral RNA and DNA. Epstein–Barr virus (EBV) establishes persistent infection by suppressing macrophage-mediated innate immune responses through regulation of the TRIM29-STING axis [28]. Furthermore, Epstein–Barr virus (EBV) targets the JAK-STAT signaling pathway, promoting the production of multiple proteins that suppress IFN-γ and Type I IFN activity [29]. Studies on HIV-1 have partially elucidated the characteristics of the antiviral responses in macrophages [30]. In M2a macrophages, HIV-1 replication is partially inhibited without impeding viral entry or reverse transcriptase activity, suggesting that suppression occurs during the late stages of the viral cycle. In contrast, in M1 macrophages, CD4 expression is downregulated, thereby preventing HIV-1 from entering the cell [30]. Indeed, because certain viral infections can benefit from macrophage polarization, more research is needed to elucidate the antiviral and immune-evasion mechanisms in macrophages clearly.

Macrophages polarize into different phenotypes (M1-like/M2-like) depending on the infecting virus. In addition, some viruses can induce complex macrophage polarization, depending on the viral strain, stage, and host. Macrophage polarization is regulated through the coordination of diverse signaling pathways, including nuclear factor kappa B (NF-κB), mitogen-activated protein kinase (MAPK), Janus kinase-signal transducer and activator of transcription (JAK-STAT), and the Notch signaling pathway. The NF-κB signaling pathway is activated by ligand engagement of Toll-like receptors (TLRs) or cytokine receptors, such as IL-1R. This activation leads to the IKK complex-mediated phosphorylation and degradation of IκBα, thereby allowing NF-κB to translocate into the nucleus. Consequently, the production of inflammatory cytokines, including IL-1β, IL-6, and TNF-α, is induced, thereby promoting polarization into M1 macrophages [31]. The MAPK signaling pathway is activated by TLR4 engagement of adaptor proteins such as MYD88 and TIRAP, leading to a signaling complex involving IRAKs and TRAF6. Subsequently, activated MAPKs such as p38 and JNK induce the expression of inflammatory cytokines and promote polarization into M1 macrophages [32]. The JAK-STAT signaling pathway regulates both M1 and M2 macrophage polarization depending on the cytokine milieu. IFN-γ activates JAK1/2 and STAT1, leading to the expression of interferon-stimulated genes (ISGs) and antiviral genes and promoting polarization toward M1 macrophages. In contrast, IL-4 or IL-13 activates STAT3/STAT6, leading to the expression of anti-inflammatory genes, such as TGF-β and IL-10, and promoting polarization toward M2 macrophages. SOCS1/SOCS3 inhibits STAT1/STAT3-mediated signaling pathways [33]. The Notch signaling pathway is activated through interactions between the Notch protein and Delta-like ligands (DLL1/DLL3/DLL4) and the Jagged family (Jagged 1/Jagged 2). Proteolytic cleavage of the protein releases the Notch intracellular domain (NICD), which then translocates to the nucleus. This signaling pathway promotes the polarization of macrophages toward the M1 phenotype and inhibits polarization toward the M2 phenotype [34, 35].

In addition, viruses have evolved various strategies to evade the antiviral response of M1-polarized macrophages, thereby creating a microenvironment conducive to viral replication within macrophages. These involve skewing polarization toward M2 macrophages by modulating the activity of inhibitory receptors, such as programmed cell death 1 (PD-1), PD-L1, and viral homologs of CD200 and CD47. PD-1 is an inhibitory receptor expressed on various immune cells, such as macrophages. It binds to PD-L1 and downregulates T cell activation by negatively regulating PI3K-Akt and Ras-MEK-ERK signaling pathways [36]. In macrophages, PD-1 inhibits STAT1 phosphorylation and enhances STAT6 phosphorylation, thereby suppressing polarization toward the M1 phenotype and promoting polarization toward the M2 phenotype [37]. Similarly, T cell immunoglobulin and mucin domain-containing protein 3 (TIM3) interacts with its ligand, galectin-9, leading to decreased STAT1 phosphorylation and increased STAT3 phosphorylation. The activated TIM3/galectin-9 signaling pathway inhibits polarization into M1 macrophages [38]. CD200R is activated upon interaction with CD200, leading to the recruitment of Dok2 and RasGAP. This process inhibits Ras-ERK and PI3K signaling pathways while activating STAT1. Viral homologs of CD200 expressed by various viruses can interact with CD200R to suppress polarization toward M1 macrophages in an NF-κB-dependent manner or to skew macrophage polarization toward the M2 phenotype [39]. CD47 interacts with signal regulatory protein α (SIRPα) on macrophages, inducing SIRPα tyrosine phosphorylation and promoting its interaction with SHP-1. This process acts as a “do not eat me” signal, inhibiting phagocytosis while simultaneously promoting macrophage polarization toward the M2 phenotype [40]. This, in turn, facilitates viral immune evasion.

Consequently, plasticity-driven polarization of macrophages acts as a key mechanism for maintaining homeostasis in response to local microenvironmental alterations [41]. Macrophages exhibit highly flexible functional transitions through various specialized subtypes following M1/M2 polarization. Following viral infection, macrophages are initially activated into the M1 phenotype, secreting TNF-α, IL-1β, IL-12, and other factors to induce a robust inflammatory response. However, if the M1 state persists for too long, the inflammatory response becomes severe enough to cause tissue damage. Subsequently, M2 macrophages suppress inflammation by secreting IL-10 and TGF-β, restoring homeostasis through tissue repair and remodeling. The balance between M1- and M2-type macrophages determines whether tissue damage or recovery occurs in the post-infectious inflammatory state. The regulatory mechanisms of this plasticity remain incompletely understood. However, the type and intensity of stimuli provided by the microenvironment have been identified as fundamental factors driving differentiation into distinct subtypes [42]. Especially the M2-like state promotes tissue protection and suppresses inflammation, although it may also create an environment that facilitates persistent infection by certain viruses [43, 44]. Therefore, the M1/M2 dichotomy alone is insufficient for understanding macrophages' dual roles. Furthermore, macrophages display heterogeneity, showing highly diverse phenotypes and functions. These findings indicate that macrophage polarization is not a fixed dichotomous state but a dynamic process involving continuous transitions. Recently, expanded polarization types beyond the M1/M2 dichotomy have been proposed. These include various intermediate/mixed/context-dependent activation states (Table 1)[45-49]. For example, M4 macrophages, differentiated by platelet-derived growth factor 4 (PDGF-4), exhibit both pro-inflammatory and foam cell-like characteristics [46]. Regulatory macrophages during the inflammation-resolution phase support tissue repair and restore homeostasis by removing necrotic cells and secreting anti-inflammatory mediators [49]. The existence of these specialized subtypes demonstrates that macrophage activity is precisely regulated by the microenvironment state. Furthermore, the functional switching of macrophages, driven by the heterogeneity and plasticity in response to the microenvironment, could be a key feature in the development of targeted therapeutic strategies for viral infectious diseases.

### Dual Roles of Macrophages in Viral Infections

**Fig. 1:**
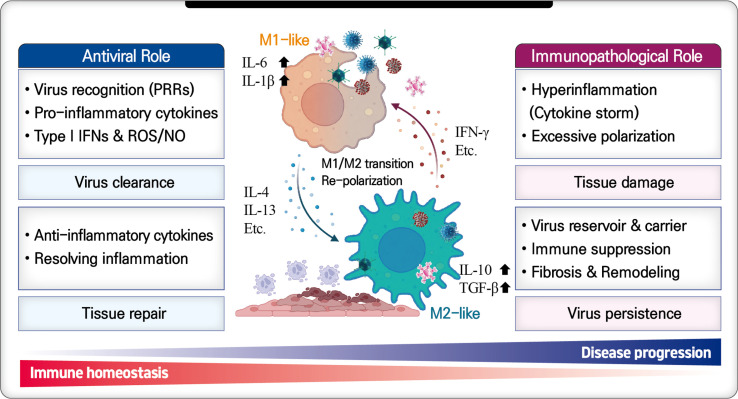
Dual role of macrophages during viral infection. Macrophages mount antiviral immune responses during viral infection, but can promote immunopathology when dysregulated. M1 macrophages play a primary role in antiviral activity by inducing a potent pro-inflammatory response; however, excessive cytokine production can result in immunopathological changes and tissue damage. Conversely, M2 macrophages promote tissue repair through antiinflammatory mechanisms, but may also facilitate viral persistent and chronic infection through immunosuppressive effects. The balance between these opposing functions is crucial for restoring immune homeostasis, determined by viral load, the stage of infection, activated immune state, and the immune microenvironment. Created with BioRender.com.

In viral infections, macrophages serve as a double-edged sword, capable of both initiating antiviral responses and causing immunopathology (Fig. 1). Understanding this dual role and adjusting macrophage activity accordingly is essential for effective antiviral strategies. Viruses exploit macrophage polarization to neutralize antiviral responses induced by M1-type macrophages or to promote polarization toward M2-type, thereby creating a microenvironment that supports their replication and spread [50]. Therefore, the level and timing of M1/M2 polarization, along with the composition of macrophage subsets at the infection site, determine whether the infection results in a protective immune response, immunopathology, or severe disease [51]. Various viruses induce polarization toward inflammatory M1-type early in infection and toward anti-inflammatory M2-type later [52]. However, pathogenic strains suppress M1 macrophage-induced antiviral responses and promote a transition to M2 macrophages to evade host defenses. In contrast, attenuated strains stimulate M1 macrophage responses, aiding in viral clearance [53]. However, the H5N1 highly pathogenic avian influenza virus (HPAIV) shows that polarization towards M1-type is beneficial for viral clearance but, if unregulated, can lead to severe tissue damage and ultimately fatal immunopathology in the host [54].

M1 macrophages produce large amounts of ROS, such as hydrogen peroxide, superoxide, and hydroxyl radicals, creating a highly oxidative environment that inhibits viral replication [55]. Specifically, NO exhibits broad-spectrum antiviral activity and therapeutic effects, including inhibition of VACV ribonucleotide reductase and targeting SARS-CoV-2's 3CL protease [56, 57]. Furthermore, M1 macrophages secrete pro-inflammatory cytokines and contribute to the antiviral responses. TNF-α inhibits the replication of viruses such as IAV, HIV-1, PRRSV, CSFV, and mouse adenovirus [58]. In HIV-1, it works by inducing RANTES and decreasing CCR5 expression [59]. In CSFV, it works by inducing NF-κB and IRF1-dependent type I IFN responses [60]. IL-6 exhibits potent antiviral effects by disrupting nucleocapsid formation and cccDNA accumulation in HBV; IL-8 suppresses HIV-1 transcription, and IL-1β synergistically inhibits HCV replication alongside IFN-α [61, 62]. IL-12 promotes Th1 differentiation of CD4+ T cells and activates NK cells to support viral clearance. Therefore, the cytokine network derived from M1 macrophages enhances antiviral responses in both innate and adaptive immunity [63]. Furthermore, M1 macrophages enhance NK cell cytotoxicity via IL-1β, IFN-β, IL-15, and other factors, whereas M2 macrophages exhibit weaker effects, resulting in differences in viral clearance efficiency [64].

Macrophages biased toward the M2 phenotype by the virus suppress the antiviral response induced by M1 macrophages. PRRSV polarizes AMs toward the M2 subtypes and effectively invades and replicates by utilizing CD163 as a functional receptor, which is highly expressed on M2 macrophages, while simultaneously suppressing the antiviral response of M1 macrophages [65]. Furthermore, RSV infection increases Gas6 expression, which interacts with Axl to suppress macrophage antimicrobial responses and promote M2 differentiation, subsequently increasing susceptibility to pneumococci and causing secondary bacterial pneumonia [66]. Thus, various viruses promote persistent infection by either exploiting strategies to enter cells via M2 macrophage-specific marker molecules or by creating an immunologically favorable extracellular microenvironment through polarization into M2 macrophages. For example, in chronic HCV and HIV/SIV infections, IL-12 production and STAT1 activation are suppressed, leading to M2-type polarization and decreased pro-inflammatory cytokine expression [52, 67, 68]. MYXV suppresses M1 polarization and promotes transition to M2 macrophages through the CD200 homolog M141R, which binds CD200R in an NF-κB-dependent manner [69]. The HCV E2 protein decreases SOCS3 phosphorylation and blocks STAT1 activation. At the same time, it increases STAT3 phosphorylation and boosts IL-10 expression, promoting M2-type polarization [70]. Furthermore, the HIV-1 Nef protein induces MHC-I degradation, impairing macrophage antigen presentation and evading CTL recognition. While VACV A35R and ASFV EP153R proteins interfere with MHC-mediated antigen presentation, suppressing T cell priming and CTL responses [71-73]. Additionally, macrophages are used for viral replication and spread. For example, ASFV mainly infects macrophages and significantly enhances viral entry efficiency by using both clathrin-mediated endocytosis and micropinocytosis [74]. Furthermore, macrophages circulate throughout the body via the bloodstream and differentiate into tissue-resident macrophages. In this process, macrophages serve as viral carriers, transporting viruses from the intracellular environment to tissues. This facilitates viral spread through cell-to-cell contact and affects the formation of co-infection or mixed-infection microenvironments. HCMV maintains a long-term, low-level productive infection in both M1 and M2 macrophages, elevating IL-6 and TNF-α levels to create a local inflammatory microenvironment. This ability to influence macrophage polarization is believed to contribute to the development of long-lasting viral reservoirs and facilitate viral spread [75].

Excessive polarization of macrophages can sometimes promote viral spread. During the acute phase of HIV infection, polarization of macrophages toward the M1-type dominates, potentially attracting more monocytes and T cells to the infection site and thus promoting HIV infection and dissemination [52]. Similarly, in severe SARS-CoV-2 infection, the inflammatory response maintained by M1-type macrophages recruited to the lungs contributes to persistent viral spread, acute respiratory distress syndrome (ARDS), and multi-organ failure [76, 77]. Meanwhile, SARS-CoV-2 triggers M2-associated gene expression in macrophages, and viral load appears low in M2-type macrophages. Therefore, SARS-CoV-2 exhibits different susceptibility and response patterns depending on the polarization state of macrophages [78].

In respiratory viral diseases, resident AMs in the lungs are generally friendly to the host. In contrast, infiltrating Mo-Macs show virus-friendly responses and are more closely associated with pathology [79]. This is believed to result from differences in the imprinting of distinct regulatory factors due to their different developmental origins [80]. Understanding these regulatory mechanisms could provide important clues for developing targeted therapeutic strategies to re-educate macrophages and improve immune defense against viruses and tissue recovery.

The function of AMs is highly depended on the local microenvironment, including contact with epithelial cells, oxygen levels, and the presence of surfactant. Consequently, they display diverse physiological roles, including pro-/anti-inflammatory, pro-/anti-fibrotic, and tissue-regenerative functions [81]. Additionally, under normal conditions, macrophages mainly show an immunosuppressive phenotype, which is essential for preventing unnecessary lung inflammation [82]. For example, CD200 expressed on the luminal surface of respiratory epithelial cells binds to CD200R on macrophages, thereby inhibiting the expression of pro-inflammatory genes and preventing excessive lung inflammation [83]. During viral infection, RTMs produce high levels of chemoattractants such as IL-1β, CCL7, and MCP-1, which quickly recruit CCR2+ myeloid-derived monocytes to the lungs. During influenza infection, AMs serve as the main producers of type I IFNs [84]. It has been reported that AMs produce higher levels of type I IFNs than pDCs, while alveolar epithelial cells produce almost no type I IFNs in response to influenza [85]. Type I IFNs trigger an antiviral response by activating ISG transcription of genes such as ISG15, IFIT1, and STAT2 through autocrine/paracrine signaling, thereby inhibiting viral replication [86]. However, not all viruses elicit a strong type I IFN response. For example, human macrophages infected with HCoV-229E showed increased secretion of TNF, CCL5, and CCL4, but there was no change in IFN-β levels [87]. Furthermore, viral infection stimulates the influx of circulating monocytes into the lungs via proinflammatory cytokines such as CCL2 and CCL3, increasing the number of defensive monocyte phagocytes and amplifying inflammation [88]. This is a crucial defensive response because viruses can impair RTM function. Indeed, in influenza-infected BALB/c mice, 90% of resident AMs were lost during the first week post-infection, whereas in C57BL/6 mice, phenotypic impairment was observed instead of a numerical decline. Both phenomena resulted in greater susceptibility to secondary bacterial infections and increased weight loss and mortality [89]. Therefore, viral infection in the respiratory tract maintains a balance between defense and damage through AM depletion and dysfunction, monocyte influx, and increased inflammation.

As mentioned earlier, AMs serve as the first line of immune defense against pathogens and foreign particles entering the respiratory tract [90], helping to maintain pulmonary homeostasis under normal conditions by exhibiting high phagocytic capacity and low production of inflammatory cytokines [81]. The pulmonary microenvironment plays a crucial role in shaping AM phenotype and function. Interestingly, reports indicate that AMs can partially exhibit characteristics of both M1 and M2 phenotypes simultaneously, depending on the nature of signals from the surrounding microenvironment, highlighting the high plasticity of macrophages. Recent studies demonstrate that resident pulmonary macrophages act as key regulators of pulmonary inflammatory responses, inducing inflammation resolution. They also play a crucial role in restoring homeostasis and initiating parenchymal tissue repair mechanisms following lung injury. Meanwhile, multiple studies utilizing bronchoalveolar lavage fluid (BALF) and surgical lung biopsies have demonstrated that macrophages also have a harmful role in causing lung lesions [91]. For example, during SARS-CoV and SARS-CoV-2 infections, the accumulation of inflammatory macrophages in tissues and dysregulation of the type I IFN response have been identified as key factors in viral immunopathology [92]. Consequently, therapeutic strategies targeting macrophages that promote and enhance SARS-CoV-2 infection have been investigated to reduce their detrimental effects [93]. Ultimately, pulmonary macrophages play a pivotal role in the immunopathology of respiratory viruses. As previously described, understanding the reciprocal regulation of macrophage functions and identifying major regulatory factors could help develop effective therapeutic strategies for viral infections.

### Macrophage-Based Therapies

Throughout immune memory, adaptive immunity produces antigen-specific memory cells that trigger strong responses upon re-exposure, while innate immunity establishes inflammatory memory that modifies subsequent responses through epigenetic mechanisms after exposure to inflammatory signals [94, 95]. Macrophages, through this innate immune memory formed after bacterial or viral infection, can influence the course of subsequent secondary diseases such as bacterial infections and cancer [96, 97]. It has been emphasized that past SARS-CoV-2 infection leaves an epigenetic imprint on the entire airway resident macrophage lineage, affecting even unrelated respiratory viral infections afterward. Following SARS-CoV-2 infection in airway resident macrophages, the activity of type I IFN-related transcription factors and the epigenetic priming of antiviral genes increase. In this process, viral pattern recognition and canonical type I IFN signaling are involved in establishing innate immune memory. This established antiviral innate immune memory subsequently boosts secondary antiviral responses during influenza A virus infection and serves as a prerequisite for reducing excessive inflammatory responses and mortality. Therefore, macrophage-based innate immune memory is antigen-nonspecific and can provide long-term cross-protection against various pathogens. It is thus regarded as a key element in developing new therapeutic strategies aimed at a wide range of pathogens [98]. Furthermore, since inflammation in viral infections is a common symptom shared by many viruses, strategies to control macrophage-driven inflammation using reprogrammed immunomodulators are regarded as an effective approach. For example, in viral infections such as SARS-CoV-2, influenza, chikungunya virus, and rhinovirus, macrophage activation and dysregulated inflammation are key factors in disease progression. In response, baricitinib has been reported to reduce the severity of COVID-19 by suppressing the expression of excessive inflammatory cytokines observed during SARS-CoV-2 infection. At the same time, levocetirizine inhibits the release of inflammatory cytokines such as IL-6 and IL-8 induced by rhinovirus infection [99, 100]. These results suggest the potential for modulating inflammatory responses in respiratory viral infections. Furthermore, dysregulation of myeloid cells and the persistence of viral reservoirs are common features in the pathophysiology of multiple viruses [101]. In particular, in HIV-1-infected non-proliferating myeloid cells, such as macrophages and related cells, virus replication is delayed, leading to the formation of viral reservoirs. This results in persistent latent virus even during antiretroviral therapy (ART). The long-term survival of these cells and ongoing inflammation are major contributors to comorbidities in PLWH, such as cardiovascular disease and neurological deficits. Therefore, macrophage dysfunction, especially excessive activation and heightened inflammation of the M1 subtype, is a common pathophysiological feature across various viral infections and is a target for antiviral therapy. Strategies to downregulate inflammation during viral infections remain under-researched, but several immunomodulators have already shown effectiveness in this area. Especially repurposing FDA-approved drugs for these uses can speed up the transition from laboratory research to clinical treatment [102]. For instance, drugs such as ruxolitinib, baricitinib, venetoclax, and dimethyl fumarate have demonstrated efficacy in controlling inflammatory responses associated with viral infections.

**Fig. 2:**
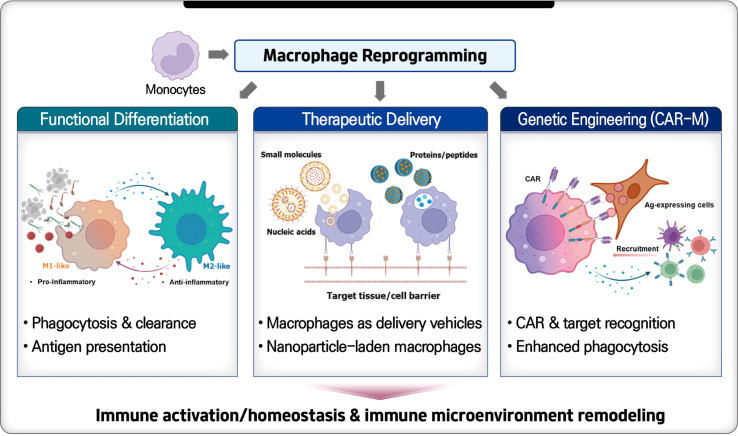
Overview of ex vivo-engineered macrophage-based therapeutic strategies. Monocyte-derived macrophages are differentiated and engineered ex vivo, then mainly applied by leveraging macrophage innate immune function, macrophage-mediated therapeutic agent delivery, and genetically engineered macrophages such as CAR-M. Created with BioRender.com.

Macrophages are used as a platform for cell therapies in various diseases due to their role in restoring tissue homeostasis and controlling inflammation. They are manipulated outside the body and then reintroduced. There are three main methods for macrophage-based treatments (Fig. 2). These include using the in vitro-differentiated macrophages' natural abilities, employing macrophages as delivery vehicles for small molecules or DNA, and using genetically modified macrophages, such as CAR-M [102]. Early therapies relied on the cytotoxicity of IFN-γ-treated primary macrophages. Recent clinical trials have shifted toward the adoptive transfer of macrophages and in vitro polarization, including CAR-M. CAR-M cells are engineered macrophages that express a chimeric antigen receptor (CAR) targeting specific antigens, allowing them to phagocytose and kill antigen-positive cells directly. At the same time, they modulate the activity of other macrophages and T cells within the microenvironment. Characterization of CAR-M activity showed that CAR-M shifts macrophage polarization from M2 to M1 within the TME and promotes T cell infiltration, thereby increasing antigen presentation mechanisms [103]. Additionally, iPSC-derived CAR-expressing macrophages (CAR-iMac) display M1 polarization and phagocytic ability [104].

Especially today, CAR-M, which combines CAR technology's targeting and effectiveness, has emerged as a new immunotherapy approach [13, 105]. CARs are receptors engineered to recognize antigens without depending on MHC. When expressed on macrophage surfaces, they replace natural receptor functions, redirecting cellular activity toward antigen-positive cells. In vivo, CAR-M reprograms the immunosuppressive microenvironment through phagocytosis, cytokine secretion, and structural remodeling. Additionally, CAR-M cells boost T cell cytotoxicity as antigen-presenting cells and coordinate comprehensive antigen-specific responses by recruiting endogenous macrophages to generate further immune effects [106]. CAR-M is also used in regenerative medicine and for the treatment of inflammatory/autoimmune diseases. Macrophages help rebuild tissues in various organs such as the lungs, heart, kidneys, liver, and intestines. CAR-M can be employed to clear neurodegenerative debris and infectious agents. Proposed functions include removing neurotoxic substances accumulated in hereditary brain disorders, reducing ECM in damaged cardiac areas, promoting intestinal epithelial cell regeneration, and eliminating excess surfactant in pulmonary alveolar proteinosis (PAP) [107, 108]. Furthermore, CAR-M cells act as powerful pro-inflammatory modulators, regulating the inflammatory microenvironment by expressing anti-inflammatory factors and cytokine receptors. This allows for control of excessive inflammation in infectious and autoimmune diseases, indicating broad potential for CAR-M [109]. However, several biological challenges remain for the clinical use of CAR-M, including maintaining long-term phenotypic stability—including stable CAR expression in hiPSCs—along with genetic engineering, cell proliferation, and scaling up manufacturing processes. Despite these obstacles, CAR-M is considered an innovative approach in immunotherapy due to its advantages, including targeted infiltration, remodeling of the immune microenvironment, and improved antigen-presenting capacity.

The currently used dichotomous classification of macrophages-M1 (inflammatory) / M2 (anti-inflammatory)-is based on biomarker expression and the functional activity of macrophages. However, it has limitations because macrophage states constantly change in response to the surrounding microenvironment, and specialized subtypes exist within each tissue [43, 110]. The markers and criteria used for M1/M2 classification differ across studies, and these markers often lack sufficient specificity. This creates challenges in developing and applying therapies targeting macrophage polarization. Consequently, the main challenge is to identify specific polarization markers to distinguish subpopulations and clarify their functions [111]. Furthermore, excessive macrophage activation can cause inflammation and worsen autoimmune diseases. Therefore, when developing macrophage-targeted therapies, it is crucial to balance effectiveness and safety [112, 113]. Recent advances in technologies such as single-cell genomics, spatial transcriptomics, and mass cytometry, combined with bioinformatics methods, are providing new insights into the diversity, adaptability, and functions of macrophages. Future macrophage-based treatments will aim to selectively remove or reprogram harmful macrophage populations, deliver targeted drugs via Trojan-horse-like mechanisms, and reintroduce differentiated or engineered macrophages from outside the body.

## Conclusion

Macrophages are essential cells of the innate immune system, involved in processes such as infection, inflammation, and maintaining homeostasis. In response to microenvironmental stimuli, they polarize into either the inflammatory M1 or the anti-inflammatory M2 macrophage subtypes, performing opposing functions. Cytokine storms mediated by macrophages during influenza and coronavirus pandemics demonstrate that dysregulation of macrophage polarization can lead to severe disease. Thus, understanding the detailed mechanisms by which viruses influence macrophage differentiation and identifying key factors that drive the functional shift of macrophage subtypes are expected to improve macrophage reprogramming and aid in the discovery of new antiviral targets.

Furthermore, the hyperactivation of M1 macrophages, commonly observed after viral infection, has become a key factor in worsening chronic/acute inflammation-related diseases. Strategies to resolve these abnormal responses have been proposed as broadly applicable therapeutic targets for various viral diseases. Macrophage-based therapies include both pharmacological treatments and ex vivo macrophage transplantation approaches. Pharmacological treatments that are currently in clinical development using repurposed drugs need further validation for safety and effectiveness. At the same time, strategies involving the transplantation of ex vivo-activated macrophages face challenges, including phenotypic instability and microenvironmental interference.

In conclusion, current research shows that macrophage-targeted therapies have already reached certain stages of clinical application, indicating their potential as part of future combination targeted immunotherapy.

## Figures and Tables

**Table 1:** Macrophage Types in specialized microenvironments.